# Hyperlipidemia in Stroke Pathobiology and Therapy: Insights and Perspectives

**DOI:** 10.3389/fphys.2018.00488

**Published:** 2018-05-15

**Authors:** Romain Menet, Maxime Bernard, Ayman ElAli

**Affiliations:** ^1^Neuroscience Axis, CHU de Québec Research Center (CHUL), Québec City, QC, Canada; ^2^Department of Psychiatry and Neuroscience, Faculty of Medicine, Laval University, Québec City, QC, Canada

**Keywords:** stroke recovery, neurovascular unit, hyperlipidemias, neurorestoration, neuroprotection

## Background

Stroke constitutes a major cause of death and disability of the adults in the industrialized world. Ischemic stroke accounts for the majority of cases (Dirnagl, 2012). Interruption of the blood supply triggers the ischemic cascade leading to cell death and inflammation (Dirnagl et al., 1999; Lo, 2008). Our understanding for the molecular mechanisms underlying neuronal death has tremendously advanced in the last decade, leading to the development of several neuroprotective agents (Moskowitz et al., 2010). Although neuroprotection was successful in experimental studies, it failed to achieve clinical benefits in acute stroke patients (Gladstone et al., 2002; Lo, 2008). Currently, tissue plasminogen activator (tPA)-induced thrombolysis is the only Food and Drug Administration (FDA)-approved treatment used in clinics to restore the cerebral blood flow (CBF) (Wardlaw et al., 2014). Nonetheless, less than 5% of stroke patients can benefit from thrombolysis, as tPA should be administered within a narrow therapeutic time window of 4.5 h after onset (Wang et al., 2004). Endovascular mechanical embolectomy has emerged as a therapeutic option when thrombolysis is unsuccessful or cannot be applied (Smith et al., 2005).

Beyond neuroprotection in the acute phase, there is a growing interest in neurorestoration that aims to promote brain remodeling in the post-acute phase (Gladstone and Black, 2000; Gladstone et al., 2002). The interest has emerged from the overwhelming experimental and clinical findings suggesting that the brain is actively trying to recover after stroke by repairing itself (Chopp et al., 2009). The neurorestorative processes include coordinated neurogenic and angiogenic responses, which aim to improve functional recovery (Ohab et al., 2006; Zhang et al., 2008; Chopp et al., 2009; Chen et al., 2014). Until now, no clinically validated neurorestorative approach exists, and cognitive/motor rehabilitation remains the only approach used in clinics in the post-stroke phase.

Evidence from clinical trials suggests that saving neurons alone after stroke may not be sufficient to develop clinically viable therapies (Gladstone et al., 2002; Lo, 2008). Neuronal survival narrowly depends upon integrity of the microvasculature, which regulates oxygen and nutrients delivery. The functional interaction between the neuronal and vascular systems is governed by the neurovascular unit, which comprises sealed endothelial cells forming the blood-brain barrier (BBB), pericytes, astrocytes, microglia, and neurons (Hermann and ElAli, 2012). As such, it is well established that any clinically viable therapies must succeed to restore the neurovascular unit by stabilizing the microvasculature, while limiting neuronal loss and stimulating neuronal plasticity. Moreover, pre-clinical investigations use essentially healthy animals, which do not adequately translate the clinical setup in which stroke patients usually present several vascular risk factors, namely atherosclerosis associated to hyperlipidemia (Gladstone et al., 2002; ElAli et al., 2011). This aspect must be adequately and systemically addressed in the pre-clinical context.

## Hyperlipidemia: an acknowledged—yet not fully understood—risk factor in stroke patients

Hyperlipidemia is caused by an excessive uptake of high-cholesterol diet leading to high levels of blood lipids. Importantly, in registries and clinical trials up to 60% of documented patients have high levels of blood lipids including cholesterol (ElAli et al., 2011). Elevated cholesterol levels (>7.0 mmol/L) are associated to an increased risk of stroke incidence (Leppälä et al., 1999). In addition to extracranial atherosclerosis, hyperlipidemia promotes cervical or coronary atherosclerosis, which predisposes to atherothrombotic and cardioembolic stroke (Ayata et al., 2013). Though hyperlipidemia is well established as a prevalent risk factor for stroke incidence, clinical investigations showed controversial results to how it influences stroke acute and post-acute outcomes. Some clinical investigations have reported a protective effect of hyperlipidemia in stroke patients, essentially via reduction of the mortality rates (Jimenez-Conde et al., 2010; Shigematsu et al., 2015). Hyperlipidemic stroke patients tend to have reduced white matter hyperintensity (WMH) volume (Jimenez-Conde et al., 2010). Severity of the WMH has been reported to predict infarct progression upon stroke leading to poor clinical outcomes (Arsava et al., 2009). Importantly, abnormal cholesterol profile in obese individuals, namely the elevated levels of low-density lipoproteins (LDL) and the reduced levels of high-density lipoproteins (HDL), has been proposed to cause white matter abnormalities (Cohen et al., 2011). This suggests that the profile of the lipoproteins is the factor to consider not only the absolute elevated levels of total blood lipids. Indeed, in a recent study it was demonstrated that LDL level was associated with long-term mortality after stroke (Xing et al., 2016). On the other hand, some other studies have shown that hyperlipidemia negatively impacts acute stroke outcomes in patients who were treated with thrombolytic agents or underwent mechanical embolectomy (Restrepo et al., 2009). Interestingly, administration of cholesterol-lowering drugs was reported to ameliorate the neurological outcomes of stroke patients (Amarenco et al., 2006). It is important to mention that most of these studies have evaluated the impact of hyperlipidemia on the long-term mortality after stroke, and important data, such as the size and location of the initial lesions were not systemically evaluated. Furthermore, it is very probable that hyperlipidemic patients might have been treated with cholesterol-lowering drugs, such as statins, before stroke incidence. For instance, several studies have indicated that patients receiving statins exhibited less severe structural injury and had better neurological outcomes (Lakhan et al., 2010). Furthermore, patients who have received statins just after stroke were more likely to be discharged home vs. patients already on statins before stroke (Moonis et al., 2014). Both group of patients were also more likely to be discharged home than those patients who did not receive statin therapy at all (Moonis et al., 2014). However, a recent study has shown that hyperlipidemia is associated to a lower risk of short- and long-term mortality after stroke irrespectively of statin use (Yeramaneni et al., 2017), thus further fuelling the controversy. Nonetheless, it is well recognized that independently of its cholesterol lowering effects, statins provide tissue protection via improvement of the microvasculature integrity, attenuation of the inflammation, and reduction of the oxidative stress (Liao, 2002; Zhao et al., 2014). Therefore, the pleotropic cholesterol lowering-independent protective effects of statins on the integrity of the microvasculature in hyperlipidemic patients should be systemically evaluated in the clinical trials in order to better interpret data (Table 1).

**Table 1:** Summary of major studies in human patients.

**Authors**	**Patients/Markers**	**Conditions/Treatments**	**Effects**
Gillman et al., 1997	Only men (45–65 years old) 1) Total fat, SFA, and MUFA 2) PUFA	NA	1) Reduced risk of ischemic stroke, TIA and hemmorrhage stroke. 2) No changes in stroke risk.
Rizos and Mikhailidis, 2001	TG, HDL, LDL, and TC	NA	a) Dubbo study: Negative association between HDL and stroke (fatal, non-fatal and TIA). No association between TG and stroke risk. b) Copenhagen study: Negative association between HDL and ischemic stroke. TG non-fasting is associated with risk of ischemic strokes. c) Finnmark study: Association between non-fasting TG and stroke only in women. d) Lowered HDL and/or raised TG levels are associated with an increased risk of cerebrovascular events. Total cholesterol, LDL, HDL and TG levels predict the risk of a cerebrovascular events.
Liao, 2002	1) High level of cholesterol and LDL-C	2) Statins	1) Impaired endothelial functions, including vasodilatation. 2) Improved endothelial functions and decreased risk of stroke.
Sauvaget et al., 2004	1) High consumption of Cholesterol and animal fat 2) SFA and PUFA	NA	1) Reduced risk of cerebral infarction and death. 2) Do not constitute a risk factor for cerebral infarction and mortality.
Amarenco et al., 2006	LDL (100–190 mg/dL)	80 mg atorvastatin/day	Decreased risk of ischemic stroke and increased risk of haemorrhagic stroke. Improved neurological outcomes after ischemic stroke.
Leppälä et al., 1999	Only men and smokers 1) HDL ≥ 0.85 mmol/L 2) Serum total cholesterol ≥ 7.0 mmol/L	NA	1) Decreased risk of subarachnoid hemorrhage and cerebral infarction. 2) Decreased risk of intracerebral hemorrhage and increase risk of cerebral infarction.
Restrepo et al., 2009	1) HL	2) Gemfibrozil	1) Exacerbated acute stroke outcomes in patients treated with thrombolytic agents or underwent mechanical embolectomy. 2) Decreased ischemic stroke risk, and TIA.
Lakhan et al., 2010	1) Total and LDL cholesterol	2) Pre-treatment with statins 3) Pre-treatment with atorvastatin	1) Do not affect haemorrhagic stroke. 2) Improved long-term neurological outcomes in Caucasian but not in African patients. 3) Improved outcomes after ischemic stroke in atherothrombotic and lacunar infarctions.
Stapleton et al., 2010	HC	NA	Increased risk of cardiovascular diseases.
Drake et al., 2011	HL, obesity and atherosclerosis	NA	Increased chronic systemic inflammation and risk of stroke.
Jimenez-Conde et al., 2010	1) Low cholesterol levels 2) HL	NA	1) Increased risk of small vessel diseases. 2) Reduced white matter hyperintensity severity in individuals with acute ischemic stroke. Decreased mortality after intracerebral hemorrhage and acute ischemic stroke. Decreased the risk of intracerebral hemorrhage and microbleeding.
Moonis et al., 2014	NA	Treatment with statins pre/post-stroke	Decreased mortality after stroke.
Park et al., 2014	TG/HDL-C ratio	NA	Associated to recurrent stroke risk.
Xu et al., 2014	High level of LDL-C, HDL-C, and total cholesterol	NA	Increased risk of acute ischemic stroke.
Zeljkovic et al., 2010	sdLDL	NA	Increased risk of mortality after stroke.
Zhao et al., 2014	NA	Statins (pravastatin 40 mg/day, atorvastatin 80 mg/day Simvastatin 40 mg/day and rosuvastatin)	Improved outcomes after stroke (oxidative stress, microvasculature integrity and inflammation). Statin pre-treatment enhanced clinical outcomes with a significant improvement in neurological deficit score.
Shigematsu et al., 2015	HL	NA	Increased sequela and hazard ratio for death on stroke.
Xing et al., 2016	LDL	NA	Associated with a long-term mortality after stroke.
Pawelczyk et al., 2017	HL (LDL and total cholesterol)	NA	Increased level of secerted P-selectin after ischemic stroke.
Yeramaneni et al., 2017	1) HL (LDL ≥ 70 mg/dL with comorbidities or ≥ 100 mg/dL without comorbidities) 2) Sweden study: cholesterol levels ≥ 178 mg/dL 3) Israeli study: cholesterol levels > 155 mg/dL	Pre-treatment with statins	1) Decreased short/long term mortality after stroke (30 days, 1 year, and 3 years). 2) Reduced risk of mortality (7 years after stroke). 3) Decreased risk of stroke severity and improve functional outcome (with/without pre-treatment with statins).
Price et al., 2018	Women Obesity/adiposity	NA	Increased risk of ischemic stroke and decreased risk of hemmorrhagic stroke (subarachnoid hemorrhage and intracerebral hemorrhage).

*NA, not-applicable; TIA, Transient ischemic attack; HL, Hyperlipidemia; HC, Hypercholesterolemia; LDL, low-density lipoproteins; HDL, High-density lipoproteins; TC, Total cholesterol; TG, Triglyceride; SFA, Saturated fatty acids; MUFA, Monounsaturated fatty acids; PUFA, Polyunsaturated fatty acids*.

## Impact of hyperlipidemia on the neurovascular unit: lessons from animal studies

In contrast to the clinical setting, there is a consensus as to the detrimental effect of hyperlipidemia in experimental stroke studies. In rodents, hyperlipidemia has been demonstrated to exacerbate the ischemic damage through endothelial cell injury, oxidative stress, inflammation and neuronal loss (ElAli et al., 2011; Ayata et al., 2013; Deng et al., 2014; Cao et al., 2015). Moreover, it has been shown that the ischemic damage in hyperlipidemic mice is directly associated to duration of the blood supply interruption (Maysami et al., 2015). It is noteworthy to mention that in the majority of these experimental studies, hyperlipidemia was mostly induced in transgenic mice lacking key genes implicated in the metabolism and transport of lipids, namely apolipoprotein E (ApoE) (Zhang et al., 1992; Zechariah et al., 2013a,b). The advantage of these mice is that they develop atherosclerosis following short-term exposure to high-fat diet, usually up to 8 weeks. In ApoE knockout (ApoE^−/−^) mice fed with a high-fat diet, hyperlipidemia has been shown to exacerbate neuronal death and loss upon ischemic stroke induction (ElAli et al., 2011; Ayata et al., 2013; Herz et al., 2014). However, in wildtype littermates fed with the same high-fat diet for the same period of time - which are often used as controls - hyperlipidemia has not significantly influenced neuronal death and loss (ElAli et al., 2011; Ayata et al., 2013; Herz et al., 2014). These results suggest that depletion of the ApoE gene might increase neuronal vulnerability to the ischemic insult independently upon the effects of hyperlipidemia. Indeed, ApoE and its receptors were demonstrated to cooperatively regulate common mechanisms essential to neuronal survival in the adult brain (Beffert et al., 2006). As such, studies involving ApoE^−/−^ mice, or other lipoprotein receptors, should be interpreted with some caution, especially when it comes to neuronal death and loss.

Hyperlipidemia has been clearly demonstrated to exacerbate vascular damage in transgenic and wildtype mouse strains. It has been shown to alter neurovascular coupling, reduce resting CBF, impair the physiologic cerebral vasodilator reflexes, and worsen cerebral perfusion deficits upon cerebral ischemia in mice even before atherosclerosis appearance in cervical or intracranial arteries (Ayata et al., 2013). Moreover, hyperlipidemia in wildtype mice, and not ApoE^−/−^ mice, has been reported to increase BBB permeability, and promote the formation of brain oedema upon cerebral ischemia in the acute phase via mechanisms implicating lipid peroxidation, matrix metalloproteinases (MMPs) activation, and RhoA over-activation (ElAli et al., 2011; Deng et al., 2014). Furthermore, hyperlipidemia has been shown to trigger an inflammatory response at the brain microvasculature associated to endothelial cell activation, and facilitate infiltration of the circulating immune cells into the brain, as well as platelet activation and adhesion to the injured endothelial cells (Stapleton et al., 2010). Indeed, positron emission tomography (PET) imaging combined to post-mortem histochemical analysis has demonstrated that hyperlipidemia is associated to microglial cell activation and over-expression of several vascular adhesion molecules on injured endothelial cells (Drake et al., 2011). These observations suggest that the detrimental effects of hyperlipidemia are mainly associated to microvasculature dysfunction, an aspect that is often neglected in the clinical setting.

It has been proposed that therapeutic angiogenesis, which aims to increase vascular density within the lesion site, should enhance the CBF and attenuate the ischemic damage (Jean LeBlanc et al., 2017). In experimental studies, therapeutic angiogenesis induced by vascular endothelial growth factor (VEGF) has been shown to promote structural and functional neurological recovery after stroke (Hermann and Zechariah, 2009). Indeed, local VEGF administration has been shown to promote the formation of functional brain microvasculature by stimulating the crosstalk between endothelial cells and pericytes (Zechariah et al., 2013a), and consequently reduced the ischemic insult severity by enhancing the CBF and stabilizing the energy state of the brain (Zechariah et al., 2013a). Importantly, hyperlipidemia has abolished the VEGF-hemodynamic improvements by disrupting the interaction between endothelial cells and pericytes (Zechariah et al., 2013b). Based on these observations, it is conceivable to postulate that hyperlipidemia could decisively influence the efficacy of strategies that aim at promoting neuroprotection and/or neurorestoration (Supplementary Table 1).

## Conclusion and perspectives

One of the priorities in the field should be elucidating the exact role of hyperlipidemia in stroke patient outcomes. One promising avenue is the detailed profile of lipoprotein subclasses. A recent study has demonstrated that the elevated levels of a specific LDL particle—small, dense LDL (sdLDL)—and not total LDL, constitute a strong predictor of stroke incidence, and most importantly is associated to an increased risk of mortality (Zeljkovic et al., 2010; Xu et al., 2014). Therefore, it might be more appropriate to investigate dyslipidemia with an emphasis on lipoprotein subclasses and their association with stroke severity and outcomes. Another major challenge in stroke therapies remains in the translational potential of pre-clinical investigations. Clinical studies were based on pre-clinical investigations obtained in healthy animals that do not have comorbid conditions (Gladstone et al., 2002; ElAli et al., 2011). As such, it is important now to systemically include animals that present at least one comorbid condition, such as hyperlipidemia, to increase the translational potential of the experimental findings. However, induction of hyperlipidemia itself is crucial aspect. It is important to avoid using transgenic animals lacking genes that could influence neuronal vulnerability to ischemic insult. As an alternative, it would be more appropriate to use non-transgenic animals fed with high-fat diet for longer time periods. Moreover, it is important to evaluate the effects of different varieties of high-fat diets. This point is important, as fat in the diets used in pre-clinical study is often prepared from well-defined and specific sources, whereas in humans the source of fat in diets is highly diversified. To sum up, it is conceivable to propose that to achieve breakthroughs in stroke therapies, pre-clinical investigations should systemically evaluate how major risk factors, including hyperlipidemia, influence structural and functional recovery while validating new neuroprotective or neurorestorative strategies.

## Author contributions

All authors listed have made a substantial, direct and intellectual contribution to the work, and approved it for publication.

### Conflict of interest statement

The authors declare that the research was conducted in the absence of any commercial or financial relationships that could be construed as a potential conflict of interest.
