# Supplementary material for: Hyperlipidemia in Stroke Pathobiology and Therapy: Insights and Perspectives
Source: Front Physiol. 2018 May 15;9:488. doi: 10.3389/fphys.2018.00488 (PMC5962805; doi:10.3389/fphys.2018.00488)
Supplement: Supplementary file 1 [file Table_1.docx]

***Supplementary Information***

**Hyperlipidemia in Stroke Pathobiology and Therapy: Insights and Perspectives**

Romain Menet^1,2^, Maxime Bernard^1,2^, and Ayman ElAli^1,2*^

^1^ Neuroscience Axis, CHU de Québec Research Center (CHUL), Québec City, QC, Canada

^2^ Department of Psychiatry and Neuroscience, Faculty of Medicine, Laval University, Québec City, QC, Canada

***Correspondence:**

Dr. Ayman ElAli

Neuroscience Axis

CHU de Québec Research Center (CHUL)

2705 Laurier Boulevard, Quebec City, Quebec

G1V 4G2, Canada

E-mail: [ayman.el-ali@crchudequebec.ulaval.ca](mailto:serge.rivest@crchuq.ulaval.ca)

**Supplementary Table 1.** Summary of major studies in animal models.

| **Authors** | **Animal species** | **Markers** | **Conditions/ Treatments** | **Effects** |
| --- | --- | --- | --- | --- |
| Zhang et al., 1992 | WT and ApoE^-/-^ or ^+/-^ mice | HC, HDL, TG, LDL, and VLDL | Diet: NS  (Without injury) | Exacerbated occlusion of the coronary artery due to arterial lesions. |
| [Horsburgh](https://www.ncbi.nlm.nih.gov/pubmed/?term=Horsburgh%20K%5BAuthor%5D&cauthor=true&cauthor_uid=10208557) et al., 1999 | Male WT and ApoE^-/-^ mice (12 weeks) | NS | Diet: RD  (17 min CCAO) | Increased neuronal damage. |
| d’Uscio et al., 2001 | Male WT and ApoE^-/-^ mice (4-5 weeks) | HC | Diet: RD or WD *(TD.88137)*  (Without injury) | Impaired endothelial cell function. |
| Methia et al., 2001 | WT and ApoE^-/-^ mice (6-8 weeks) | LDL | Diet: RD or WD *(TD.01021)*  (Cold-induced injury) | Increased BBB permeability. |
| Chen et al., 2003 | Wistar rat (270-300g) | Statins (atorvastatin and simvastatin) | Diet: NS  (2 h intraluminal MCAo) | Improved functional recovery, associated to enhanced angiogenesis, neurogenesis, and synaptogenesis in low dose but not in high doses. |
| Hayakawa et al., 2007 | Male ICR mice (6 weeks) | HC | Diet: RD, HFD *(F2HFD1)* or CR  (2 h intraluminal MCAo) | HC increased whereas CR decreased cerebral infarction. |
| Kitayama et al., 2007 | Female ApoE^-/-^ or ^+/-^) mice (7-13 months) | HC | Diet: RD or HFD *(TD.03159)*  (Without injury) | Increased endothelial dysfunction in cerebral arterioles. |
| [Nagai](https://www.ncbi.nlm.nih.gov/pubmed/?term=Nagai%20N%5BAuthor%5D&cauthor=true&cauthor_uid=17596132) et al., 2007 | Male WT, PAI^-/-^, and Lep/ob mice | LDL, and HDL | Diet: Standard fat diet *(kM-04-k12)* or HFD *(TD.88137)*  (Permanent photochemical MCAo) | Increased ischemic injury and oedema. |
| Terao et al., 2008 | Male WT, ob/ob, and ob/ob+lep mice | HC | Diet: NS  (30 min intraluminal MCAo) | Increased BBB permeability and oedema. |
| Drake et al., 2011 | Male rats (obese, atherosclerotic and insulin resistant), and ApoE^-/-^ mice | Atherosclerosis, HL, and obesity | Diet: RD and atherogenic diet *(18.5% fat)*  (Without injury) | Increased chronic systemic inflammation and post-stroke structural damage. |
| ElAli et al., 2011 | Male WT and ApoE^-/-^ mice (6 weeks) | HL | Diet: RD or WD *(TD.88137)*  (30 min MCAo) | Increased BBB permeability and brain oedema. |
| Ayata et al., 2013 | Male WT and ApoE -/- mice | HL, LDL, and HDL | Diet: RD or WD *(42% of total calories from fat and 0.15% cholesterol)*  (1 h MCAo) | Disrupted cerebral blood flow perfusion. |
| Li et al., 2013 | Male Wistar rats | TG, and TC | Diet: RD or HFD *(45% fat)*  (3 h intraluminal MCAo) | Increased infarct size and mortality rate. |
| Takechi et al., 2013 | Female WT mice (6 weeks) | TG, and TC | Diet: RD *(AIN93M)*, RD+SFA *(cocoa butter)*, or cholesterol *(1% AIN93M)*  (Without injury) | Increased risk of BBB dysfunction. |
| Zechariah et al., 2013 | Male WT and ApoE^-/-^ mice (9 weeks) | HL | Diet: RD or WD  (90 min MCAo using microvascular clips) | Increased ischemic injury via decreased BBB integrity and altered angiogenesis. |
| Deng et al., 2014 | Male MMP9^-/-^ mice (6 weeks) | NS | Diet: HFD *(45 % calories, D12451)* or RD  (90 min intraluminal MCAo) | Altered vascular remodelling, and worsened neurological outcomes. |
| Herz et al., 2014 | Male WT and ApoE^-/-^ mice | HC | Diet: RD or WD *(TD.88137)*  (20 min intraluminal MCAo) | Increased ischemic injury and associated to exacerbated peripheral and brain immune responses. |
| Cao et al., 2015 | Male rats (250-300g) | HL, TG, TC, HDL-C, and LDL-C | Diet: *HFD (10 % lard)*  (2 h intraluminal MCAo) | Increased oxidative damage, inflammation and neuronal apoptosis. |
| Maysami et al., 2015 | Male mice (8 weeks) | Obesity | Diet: RD or HFD *(60% fat)*  (20-30 min intraluminal MCAo) | Altered inflammatory responses negatively impacting outcomes. |
| Kraft et al., 2017 | WT and Ldlr^-/-^ mice (6 or 12 month) | HC, and LDL | Diet: Atherogenic diet *(15% milk fat)*  (Without injury) | Increased thrombotic occlusions and microhaemorrhages. |

NS, Non-specified; MCAo, Middle cerebral artery occlusion; CCAO, Common carotid artery occlusion; HL, Hyperlipidemia; HC, Hypercholesterolemia; LDL, low-density lipoproteins; HDL, High-density lipoproteins; TC, Total cholesterol; TG, Triglyceride; HFD, High-fat diet; RD, Regular diet; WD, Western diet; CSVD, Cerebral small vessel disease; CR, Caloric restriction; SFA, Saturated fatty acids; BBB, blood-brain barrier.

**References**

Zhang, S.H., Reddick, R.L., Piedrahita, J.A., and Maeda, N. (1992). Spontaneous hypercholesterolemia and arterial lesions in mice lacking apolipoprotein E. Science 5081, 468-471.

Horsburgh, K., Kelly, S., McCulloch, J., Higgins, G.A., Roses, A.D., and Nicoll, J.A. (1999). Increased neuronal damage in apolipoprotein E-deficient mice following global ischaemia. *Neuroreport* 10, 837-841.

d'Uscio, L.V., Smith, L,A., and Katusic, Z.S. (2001). Hypercholesterolemia Impairs Endothelium-Dependent Relaxations in Common Carotid Arteries of Apolipoprotein E-Deficient Mice. *Stroke* 32, 2658-2664.

Methia, N., André, P., Hafezi-Moghadam, A., Economopoulos, M., Thomas, K.L., and Wagner, D.D. (2001). ApoE Deﬁciency Compromises the Blood Brain Barrier Especially After Injury. *Mol. Med.* 7, 810-815.

Chen, J., Zhang, Z.G., Li, Y., Wang, Y., Wang, L., Jiang, H., et al. (2003). Statins induce angiogenesis, neurogenesis, and synaptogenesis after stroke. *Ann. Neurol.* 6, 743-751. doi: 10.1002/ana.10555

Hayakawa, K., Mishima, K., Nozako, M., Hazekawa, M., Aoyama, Y., Ogata, A., et al. (2007). High-cholesterol feeding aggravates cerebral infarction via decreasing the CB1 receptor. *Neurosci. Lett.* 414, 183-187. doi: 10.1016/j.neulet.2006.12.022

Kitayama, J., Faraci, F.M., Lentz, S.R., and Heistad, D.D. (2007). Cerebral Vascular Dysfunction During Hypercholesterolemia. *Stroke* 38, 2136-2141. doi: 10.1161/STROKEAHA.107.481879

Nagai, N., Van Hoef, B., and Lijnen, H.R. (2007). Plasminogen activator inhibitor-1 contributes to the deleterious effect of obesity on the outcome of thrombotic ischemic stroke in mice. *J. Thromb. Haemost.* 5, 1726-1731. doi: 10.1111/j.1538-7836.2007.02631.x

Terao, S., Yilmaz, G., Stokes, K.Y., Ishikawa, M., Kawase, T., and Granger, D.N. (2008). Inflammatory and injury responses to ischemic stroke in obese mice. *Stroke* 39, 943-950. doi: 10.1161/STROKEAHA.107.494542

Drake, C., Boutin, H., Jones, M.S., Denes, A., McColl, B.W., Selvarajah, J.R., et al. (2011). Brain inflammation is induced by co-morbidities and risk factors for stroke. Brain Behav Immun. 6, 1113-1122. doi: 10.1016/j.bbi.2011.02.008

ElAli, A., Doeppner, T.R., Zechariah, A., and Hermann, D.M. (2011) Increased blood-brain barrier permeability and brain edema after focal cerebral ischemia induced by hyperlipidemia: role of lipid peroxidation and calpain-1/2, matrix metalloproteinase-2/9, and RhoA overactivation. Stroke 42, 3238-3244. doi: 10.1161/STROKEAHA.111.615559

Ayata, C., Shin, H.K., Dileköz, E., Atochin, D.N., Kashiwagi, S., Eikermann-Haerter, K., et al. (2013). Hyperlipidemia disrupts cerebrovascular reflexes and worsens ischemic perfusion defect. *J. Cereb. Blood Flow Metab.* 6, 954-962.

Li, W., Prakash, R., Chawla, D., Du, W., Didion, S.P., Filosa, J.A., et al. (2013). Early effects of high-fat diet on neurovascular function and focal ischemic brain injury. *Am. J. Physiol. Regul. Integr. Comp. Physiol.* 304, R1001-1008. doi: 10.1152/ajpregu.00523.2012

Takechi, R., Galloway, S., Pallebage-Gamarallage, M.M., Lam, V., Dhaliwal, S.S., and Mamo, J.C. (2013). Probucol prevents blood–brain barrier dysfunction in wild-type mice induced by saturated fat or cholesterol feeding. *Clin. Exp. Pharmacol. Physiol.* 40, 45-52. doi: 10.1111/1440-1681.12032

Zechariah, A., ElAli, A., Hagemann, N., Jin, F., Doeppner, T.R., Helfrich, I., et al. (2013b). Hyperlipidemia attenuates vascular endothelial growth factor-induced angiogenesis, impairs cerebral blood flow, and disturbs stroke recovery via decreased pericyte coverage of brain endothelial cells. *Arterioscler. Thromb. Vasc. Biol.* 7, 1561-1567. doi: 10.1161/ATVBAHA.112.300749

Deng, J., Zhang, J., Feng, C., Xiong, L., and Zuo, Z. (2014). Critical role of matrix metalloprotease-9 in chronic high fat diet-induced cerebral vascular remodelling and increase of ischaemic brain injury in mice. *Cardiovasc. Res*. 4, 473-484. doi: 10.1093/cvr/cvu154

Herz, J., Hagen, S.I., Bergmüller, E., Sabellek, P., Göthert, J.R., Buer, J., et al. (2014). Exacerbation of ischemic brain injury in hypercholesterolemic mice is associated with pronounced changes in peripheral and cerebral immune responses. *Neurobiol. Dis*. 456-468. doi: 10.1016/j.nbd.2013.10.022

Cao, X.L., Du, J., Zhang, Y., Yan, J.T., and Hu, X.M. (2015). Hyperlipidemia exacerbates cerebral injury through oxidative stress, inflammation and neuronal apoptosis in MCAO/reperfusion rats. *Exp. Brain Res.* 10, 2753-2765. doi: 10.1007/s00221-015-4269-x

Maysami, S., Haley, M.J., Gorenkova, N., Krishnan, S., McColl, B.W., and Lawrence, C.B. (2015). Prolonged diet-induced obesity in mice modifies the inflammatory response and leads to worse outcome after stroke. *J. Neuroinflammation* 12, 140. doi: 10.1186/s12974-015-0359-8

Kraft, P., Schuhmann, M.K., Garz, C., Jandke, S., Urlaub, D., Mencl, S., Zernecke, A., et al. (2017). Hypercholesterolemia induced cerebral small vessel disease. *PLoS. One* 12, e0182822. doi: 10.1371/journal.pone.0182822.
